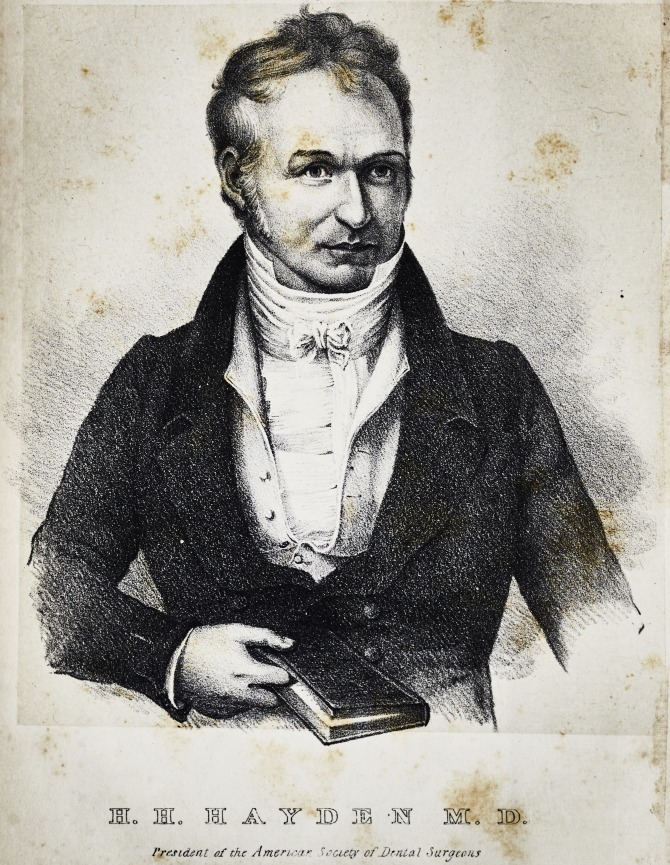# Opening Address

**Published:** 1841-09

**Authors:** H. H. Hayden

**Affiliations:** President of the Society.


H. H. HAYDEN M. D.

*President of the American Society of Dental Surgeons*

For the American Journal & Library of Dental Science

*From an original Painting by Rembrandt Peale. 1825.* *Lithogr. by Ed Weber & Co. Baltimore.*

THE AMERICAN
### JOURNAL AND LIBRARY
OF
### Dental Science.

Vol. II.] SEPTEMBER, 1841. [No. 1.

[Extracts from the Transactions of the Second Annual Meeting of the American Society of Dental Surgeons, held in the city of Philadelphia, August 10th, 11th, and 12th, 1841.]

### ARTICLE I.

*Opening Address.* Delivered by Professor H. H. Hayden, M. D., D. D. S., President of the Society.

Amidst the numerous and diversified subjects that present themselves for our contemplation, as embraced in the moral and physical sciences, no one, it would seem, need hesitate long in selecting one as best suited to the present occasion, or as affording, not only a temporary amusement and edification, but of rational entertainment. Not so, however, with myself in the present instance, for I find that I am placed in a novel situation.

Had I been requested to deliver an address to some long established literary or scientific society—or on some particular subject, either civil, religious, or political—had I been called upon to address you upon the religion of the Hindoos, or that of the aborigines of our own country—upon the legal codes of Lycurgus, and the wonderful depth of human sagacity and forecast manifested therein—or upon the subject that has called forth the wonder and astonishment of the admiring nations of the whole civilized world—the constitution of the United States, I should feel as if I had something substantial to stand or build upon.

But far different is my situation in the present case—I am deputed to deliver an address to whom? or to what? To a self-created body, which thirteen months ago was not known or acknowledged as belonging to any society or community—without a legitimate head or name, without laws, without government, and, with some exception, without character, except that of the mercenary and unprincipled. But which, from a deep sense of the hitherto unpropitious state and condition of the highly deserving members of the profession, which we have hitherto, and do still wish to cultivate and preserve, have ventured, in accordance with a deep and abiding sense of the propriety of the course, assumed a position or stand, which, if encouraged and sustained, will insure to us not only a respectful but a legitimate claim to the title of zealous cultivators of science, and the undeviating friends of humanity and of social order.

Such is the position, and such the light in which, under existing circumstances, we will consider ourselves in the present instance.

But one short year since, gentlemen, we ventured upon the novel enterprise of convening in a neighbouring city, where the generous and liberal spirit of our professional brethren greeted us with an unlooked for and encouraging welcome, to form, or rather to lay the foundation of a society, or community of dental surgeons.

But few, we presume, that were present on that occasion, will hesitate to acknowledge the fears and apprehensions entertained lest the object of our efforts, however laudable, useful, or commendable they might prove, if successfully carried into operation, might prove abortive, or end in defeat and discomfiture; and be it known and freely acknowledged, that but for the noble, generous and apparently disinterested zeal of the members of that city, and others of the profession, convened on the occasion, we should have been spared the trouble, and deprived of the pleasure of meeting here this day, to receive and reciprocate the hearty welcome, and of uniting our efforts in carrying out, adorning and perfecting the superstructure, which was but imperfectly accomplished at our last meeting.

On that occasion, notwithstanding the unlooked for, nay, the unexpected difficulties which were thrown in our way, and which we *had* to encounter, we succeeded in accomplishing more than we had a right to expect; for be it known, that there were not wanting those who like Samballat, when the Israelites attempted to rebuild the broken and mutilated walls of Jerusalem, said, "What do these feeble Jews? Will they fortify themselves? Will they sacrifice? Will they make an end in a day? Will they revive the stones out of the *heaps* of *rubbish* which are burnt?" And others, like Tobiah, the Ammonite, who said upon the same occasion, "Even that which they build, if a fox go up he shall even break down their stone-wall." Nehemiah, iv, 2, 3. Intimating that even *our* attempt to form a society could not, or would not stand or succeed.

Fortunately, these uncalled for and unseasonable prognostications have not, as yet, been verified or fulfilled. On the contrary, our society was formed—has continued to prosper—to be encouraged by the eulogies and encomiums of the medical philosophers, of not only our own, but even of those of foreign countries and the societies thereof.

On this day we have again convened, not to form a society, but to carry out, and if practicable to perpetuate, the rules and regulations of the constitution and by-laws by which we are to be governed, under the sanction of legislative enactment, and to execute the plans which were contemplated for the promotion of the welfare and prosperity of the society thus formed, and for the elevation of the character of the profession which we have espoused.

Having been thus formed and organized—having been thus convened on the anniversary of the formation of the society, three points present themselves for consideration, and for our deliberation, viz:—1. What terms are best calculated to answer the purpose of an appeal to your feelings, or to your zeal in your efforts to cherish and promote the objects of an institution so novel, so laudable, and so important in its ultimate results?

2. What position or rank shall we assume, with the probability of being recognized as co-workers in the cause of science?

3. What subject or what theme shall I, or can I introduce on this occasion, that is best calculated to elicit and engage your attention—to amuse and entertain—but, more especially, to enlarge the sphere of knowledge or to edify?

In reply to the first point it would seem that no one need be at a loss to determine that a simple, unadorned statement of correlative facts, that have an intimate bearing and connection with the subject in which we are engaged, will be, if not the most agreeable, at least the most profitable and instructive in the end.

2. What rank ought we, or shall we assume?

In reply to this interrogatory, we may observe that although not hitherto recognized as being entitled to a niche in the temple of Esculapius, we assume the title, and claim the rights and privileges of being the studious, diligent and successful cultivators of at least a branch of that important, noble, and only divinely sanctioned science that was ever pursued and cultivated by man—the science of medicine.

The subject, then, which presents itself as being most appropriate or seasonable for the present occasion, is that which has a bearing upon, and an intimate relation with the science of medicine.

So far as relates to our department, its relation to, and connection with the science of medicine, it may not be considered as to its antiquity as coetaneous with the general subject, nevertheless co-existent and inseparable in its connection and its principles, as universally acknowledged. In order then to a *full* and comprehensive view of the subject which necessarily embraces the origin and progress of the science of medicine, it would seem not only proper, but requisite, to give a concise but comprehensive history of the origin and progress of the science from the commencement to the present time.

Such a course would, doubtless, prove highly entertaining and instructive to many that are present; but we claim not the talent for the performance of a duty so arduous and so interesting, and much less so the time necessary for its accomplishment.

We may venture, however, with a view to determine the relative position in which we stand, as a co-ordinate branch, engage in a brief sketch of its history, in order to substantiate our legitimate claim, not only to relationship, but in order to identify ourselves with the parent stock.

Having indulged in our remarks thus far, we will proceed to notice more particularly the object of our pursuits—its origin—its intimate connection with the general science—of its progressive improvements, and of its present state and condition as a branch of the medical science.

Of the various departments of science, that of medicine, with its auxiliary branches, whether considered in relation to its magnitude and importance, of its intricacies, and the infinitely varied modifications of which it is susceptible, or of its utility in alleviating the distresses, and administering to the comforts of the human family, may be said to stand decidedly pre-eminent to that of any other that has ever engaged the study and pursuits of man.

Not only may it be considered as holding a pre-eminence over that of any other in point of importance and utility, but also in point of antiquity it may be said to stand distinguished and without a rival—for inasmuch as disease and death were entailed upon our first parents and their lineal descendants, we may reasonably infer that the healing art has been held in requisition, and perhaps practised, though for a long time in a rude and uncultivated manner, from the birth of Cain and Abel to the present time.

It is not the object or intention of this essay, however, to establish the period of time at which the healing art was first known, or by whom first cultivated and practised, much less is it our intention to trace up in regular gradation, the important discoveries and successive improvements that have been made since the subject has been methodically and systematically treated, and as such constituting the first in the circle of sciences, and to which almost all others are directly or indirectly tributary—neither is it consistent with our present plan to enumerate or point out the names of all those who have signalized themselves as the votaries of science, and whose lives have been devoted to the rearing and embellishing of this splendid fabric. It will suffice to observe, that from the earliest periods of time to the present, disease has been one of the inseparable concomitants of human existence. That from an increase of population, and a variety of general or local circumstances, over which, either through ignorance or a want of attention, human skill had but little or no control, disease, though under simple forms, became more frequent, and more generally prevalent. Moreover, in every age and nation, we find that in proportion as the inhabitants of the earth were disposed to relinquish their simple customs and manners, as habits of civilization and refinement were inculcated amongst the human family; in proportion as a taste for luxury and ease, gained an ascendancy over reason and judgment, so much as to become the predominant, or ruling passion, so do we find disease in some of its varied forms, as sure attendant in the train, and progressively assuming a more general and extensive sway.

Such a state of things was necessarily calculated to arouse the genius of the guardians of health—to awaken and call forth the energies of their skill in devising means for the mitigation of human suffering, and to control, if possible, the prevalence of diseases that were daily consigning to the silent, the cheerless mansions of the tomb, the fairest and the noblest works of creation.

In the pursuit and cultivation of this important science, scarcely had the practicien of early times become initiated in his profession, and made familiar with the more common and simple forms of disease, when new and anomalous cases were frequently presented to his view, and demanding the exercise of new, and as yet, untried skill.

In an age of ignorance, when neither the attention nor views of the practicien were directed either to the remote or proximate causes of disease—when, it can scarcely be said that any degree of responsibility was attached to the profession, we may reasonably conclude that the treatment was mostly rude, unskilful, and most frequently unsuccessful. But the practicien, being ambitious of a name and character for skill, and stimulated by defeat, feels himself urged on to greater vigilance, and more powerful efforts for the accomplishment of his purpose in subduing the disease and restoring the health of his patient. Hence they were induced to examine, not only the ordinary symptoms of disease, but the various types and modifications of which it is liable under different circumstances, and in various parts of the human system.

This course, so well calculated to instruct and edify, soon opened a widely extended and fruitful field for the exercise of talent and the advancement of the medical science; and in a careful investigation of the subject, it was soon found that not only the human body was liable to disease, both of a general as well as local nature, but that the several parts composing the system, and destined to different specific offices, were liable to diseases *peculiar to themselves,* each requiring a treatment adapted to the particular exigencies of the case. Thus it was found that the skin, the cellular substance, the muscles and the bones, in particular, were liable to diseases of a specific character. The pelvic viscera with its appendages, the abdominal and thoracic viscera, with their appendages, were likewise accompanied with morbid phenomena peculiar to themselves. The head, viz. the greater and lesser brain, with their appendages, the ear and the eye, were no less the seat of disease, often fatal to the part, and even to life itself. Moreover, the mouth, with its appendages, particularly the teeth and the surrounding parts, though last, yet not the least in importance, so far as relates to the *results,* is often the seat of disease, causing great disturbance in the general system, and which likewise, in some cases, terminates in the death of the patient.

Hence, as the knowledge of the science advanced, and a zeal for its improvement and cultivation was manifested, these several parts, with their diseases, became the subject of critical investigation and of discussion. Hence the origin of numerous, vague, and often contradictory opinions, and various treatises intended to elucidate and explain more satisfactorily the strangely diversified, and no less complex characters which were occasionally presented to view.

It is unnecessary to enumerate, and equally as much so to particularize the various discoveries and improvements that were made in the healing art in the earlier periods of its existence, or of its progress through subsequent ages. It is sufficient to state that, although the various diseases incident to the several parts of the human body generally, were, for an immense lapse of time, under the exclusive cognizance of the physicians or medical practicien, it was found that the science of medicine was not only susceptible of subordinate divisions, but that in order to promote a more general and correct knowledge of physiology and pathology, it was highly expedient that the several parts of the human body, with the diseases peculiar thereto, should be more methodically and distinctly investigated, and separately treated of. Hence we have the following branches—viz. general, descriptive, and pathological anatomy—surgery, general and critical—practice of medicine—obstetrics, or midwifery—ophthalmic surgery—aurist—and moreover, that branch which falls under the denomination of dental surgery, the subject to which I beg leave to invite your particular attention during the present discourse.

Hitherto we have indulged in a superficial sketch, a brief outline of the probable origin and gradual progress of the healing art, or as it may perhaps be more properly termed, science of medicine, which, from the commencement, embraced each and every ailment which the human body is, or has been heir to; consequently including those connected with, or resulting from a disordered condition of the teeth; and that too without having included the latter in the view. It is not, however, to be inferred that no notice was taken of the fact, or efforts made to alleviate the sufferings consequent upon a defective condition, or diseased state of those organs. By no means; we have satisfactory proof that the attention of the earliest practiciens was directed to the diseases of the mouth; yet without any method, or system, formed upon a correct physiological, or pathological knowledge of the parts. Hence we are unwilling to admit for a moment, that our profession had even an existence as a branch of the general science, in those early periods of its practice. We are fully aware of the attempts that have been made to trace the origin of our profession to the highest and most remote antiquity, by asserting that vestiges of the art have been found amongst the Hebrews, the Egyptians, the Greeks, and Romans. These remains, or specimens of the art which are mentioned by several of the poets, are doubtless the rude efforts or attempts to supply, by the assistance of a little mechanical skill or ingenuity, the loss of several teeth, even by carving them out of wood or bone with a simple penknife. This we have known to have been done, and that too with no small degree of success by a common unlettered stone-cutter.

These specimens, or possibly highly valued relics, may have been found carefully preserved, and also some rudely formed instruments with which the teeth were made; but it by no means proves that the persons by whom such specimens were executed, could have entertained, at that period of time, the most distant idea that the defects which they were endeavouring to supply, had any relation to, or connection with, the subject as a branch of the medical science; no more than an attempt, by a lady or gentleman, at the present day, to attempt to supply a defect of the kind by the use of white wax. Of this we are fully satisfied by the fact. 1st. That at that period of time the knowledge of anatomy and physiology of the human system was not known, nor could be obtained until even a much later period of time. 2d. Although attempts were made and practised to control certain affections of the mouth, by the earliest practiciens, yet no *correct* physiological or pathological views of this branch of the science could have been had, until the phenomena of the circulation of the blood was made known by the untiring but successful researches of the immortal Hervey.

It would doubtless be not only gratifying, but somewhat edifying, to many who may be present, to listen to a critical historical essay, descriptive of the origin, progress, and present condition of our branch of the general science, with the names of those who have signalized themselves by the skill and perseverance evinced in their labours to perfect the science. But this course would be utterly incompatible with the plan contemplated, and with the time allotted for the duties incumbent upon us. Suffice it to say, that the time when the knowledge of our branch of the medical science began to attract notice and to be cultivated, was in the days of Hippocrates, about four hundred years before the christian era. To this great luminary, the reputed father of medicine, we are mostly indebted for the first intimation of a connection existing between the diseases of the body, and such as are primarily seated in the mouth.

This descendant of Esculapius, says an eminent writer, contributed largely to the promotion and advancement of the healing art in all its parts. To the knowledge which had been transmitted to him, he added that of extensive practice, and his no less extensive experience. "His attentive eye was attracted to every malady, and no symptom that accompanied disease was suffered to pass unnoticed or unexamined, even those of the teeth were made the subject of critical inquiry." The important hints and maxims which he left, and which are mostly embodied in his aphorisms, were found so essentially correct and true, that they were not only advocated and sustained, but confirmed by the practice and experience of his immediate disciples and successors, amongst whom the most eminent, perhaps, were Galen and Ætius, the former the great admirer of his genius and his works, the latter, no less an admirer of his extraordinary sagacity and professional skill.

When we reflect upon the extraordinary talents of Hippocrates, the age in which he lived, the uncommon display of skill manifested in his professional practice, and the invaluable legacy which he left to posterity in his aphorisms and other voluminous works, but above all upon the seemingly providential manner in which these works have been preserved through the long protracted ages of mental darkness, or stupid ignorance, to serve as unerring guides, or theoretical maxims, to the practiciens of the present time, in which neither their force or energy is lessened, nor their truth and correctness in the least diminished; we are lost in amazement, and ready to acknowledge that, with the exception of the records of Holy Writ, but few examples or parallel cases are left for our consideration.

It is by no means in accordance with the duty prescribed to us in the present instance, to indulge in a train of remarks upon the character and talents of this great man; but we claim your indulgence while we transcribe a few appropriate lines from a review of Dr. Marks' edition of Hippocrates, which is as follows, viz: "More than 2200 years have elapsed since the existence of Hippocrates, yet his writings have all been preserved entire *through* ages of revolutions and subversions of empires. The precious legacy to mankind, of his *wisdom* and *precepts,* has escaped the destructive hand of time, of *devastating barbarous invaders* and conquerors. Again of his works: "It was devised by consummate wisdom, dictated by long experience; the *facts* and observations it embraces are so fundamental, that they have proved an *unerring guide* in *all* ages, in *all* countries; hence *all* nations have, in common concert proclaimed them as *auricular axioms* of the healing art. Med. Rep. 7, hexad. 2, vol. 1, p. 265.

We now, for the sake of brevity pass over that gloomy waste in which science and literature languished, and the arts of civilization and refinement became almost extinct, and make an effort to collect the scattered facts that are calculated to afford us light and assistance in the further investigation of our subject.

We find then, that notwithstanding the low and discouraging state and condition of the arts and sciences, and especially that of medicine, during several centuries, the precepts of Hippocrates, of Galen, of Ætius, were still adhered to and practised in the various diseases of the mouth, and such as originated from, or depended upon the teeth. But enjoying, as they did those advantages, a very long interval of time elapsed between that of Hippocrates and Celsus, in which very little knowledge was acquired, or improvement made in the treatment of the diseases of the mouth; or if otherwise, but slight mention has been made of the subject, except of the various preparations that were employed, by different persons, for cleansing and preserving the mouth and teeth healthy and clean, such as powders, electuaries, elixirs, &c. &c.

In the eleventh century we find that Albucasis, an Arabian physician of eminence in that day, has given some details of operations, rather mechanical than otherwise, that were practised for the relief and comfort of suffering patients.

From, and anterior to that period, this as well as almost every other branch of the medical science, was considered as appertaining *exclusively* and of *right* to the medical practicien.

About the latter end of the fifteenth century, or the commencement of the sixteenth, the whole science as well as practice of medicine, was mostly left to the tender mercies of illiterate barbers, or to the capricious whims and visionary dreams of astrological mountebanks.

During the whole of the sixteenth century, much was done in clearing away the superstitious dogmas of alchymists, and the no less absurd notions of those who advocated the unity of disease, and the discovery of an universal panacea, or specific, for every disturbance which the human body is heir to.

Whilst the general science was making slow but manifest advances, and zealous efforts were made by a number of medical writers to improve the science, our own branch was by no means left out of view; but in connection with the general subject, was treated of by several, even in the latter part of the fifteenth century.

In most of the subsequent writers, and in those of the sixteenth century, and more especially the latter part, although no systematic work was written exclusively on our profession, no less than thirty-eight treatises, or works, were written and published, mostly by medical writers on diseases of infants during the period of dentition of the mouth, and the adjacent parts. In many of these, interesting and important facts were elicited; but as the authors, at that period, were mostly ignorant of the correct physiology of the teeth, and the surrounding parts, and the phenomena accompanying their development, no rational conclusions, or positive deductions could be expected while the subject remained veiled in obscurity; of course, all efforts at the treatment were the result of conjecture, consequently, seldom exempt from error.

The science, however, in every department, was still advancing, and at or about the commencement of the seventeenth century, the branch of dental surgery began to assume a prominent rank, as one requiring a separate and distinct management.

It seems proper to observe here, that in the view that has been taken, it is, by no means, intended that up to the period last mentioned, the science of medicine had previously remained in darkness, and that no notice or mention had ever been made of any thing connected with our profession. Far from it—it is intended to refer to that period when almost all the arts and sciences were enveloped in darkness, while the invaluable precepts of ancient writers lay buried amongst the rubbish of heathenish superstitions, unheeded and forgotten. Amongst these, we ought not, nor cannot leave out of view the precepts, and aphorisms of that great luminary, Hippocrates, the justly styled father of medicine, as already mentioned.

We shall now resume the subject as connected with that of dental surgery, which, as we have observed, in the latter part of the sixteenth century, but more particularly in the beginning of the seventeenth century, began to assume a different and more interesting aspect; so much so at least as to have engaged the attention of many eminent medical writers, naturalists, and particularly some whose pursuits have been directed to the treatment of the diseases of the mouth and teeth; amongst these was Laudemiey, of Paris, who from the knowledge he had acquired, and the celebrity which he enjoyed, was sent for to the court of Spain, in the year 1716, to perform a small operation upon the mouth of Philip V. then sovereign of that kingdom.

We are not aware that Laudemiey had ever written, or intended to write upon the subject of his profession; but we find that in 1723, he was acting in the capacity of dental surgeon to his Catholic majesty, Philip V. king of Spain.

At that period we also find, that Fauchard, a dental surgeon of Paris, was engaged in writing a work on the subject, and which was published in 1728, in two volumes; and in which was made the *first* attempt to systematize and treat methodically, the dental art, as a distinct and separate branch of the medical science;—hence he has ever since been considered, and justly styled the "Father of Dentists."

The ample and highly interesting views which Fauchard had taken, and the conclusions which, from long experience he had arrived at, seemed to present a new and widely extended field for exploration, and which was not long suffered to remain uncultivated; for such was the interest which his views had excited, and such their importance, when considered in connection with the welfare and comfort of human beings, that many were soon enlisted in the cause, with a zeal and perseverance scarcely equalled, and, perhaps, never surpassed by those who were engaged, at the same time, in an investigation and experiments in the higher branches of medical science: of such were a Bunon, a Tenon, Bourdet, Mouton, &c.; who with untiring patience, and for a long time were engaged in the great hospitals of Paris, where they were free to indulge in post mortem examinations, to the full and entire gratification of their curiosity, or that of their most earnest desires.

Few, very few indeed, have contributed more to the fund of professional knowledge, or have done more to enlarge the sphere of pathological information than these men.

We would, with peculiar pleasure, extend our views, and bring into bold relief, the names of others who have been alike co-workers in building up and establishing the character of our profession as a *legitimate* branch of the medical science; but the shortness of time, and the fear of trespassing too freely upon your patience, must be plead our apology.

It has been remarked, that up to the year 1716, no less than thirty-eight different works were published on this branch of medical science, and in the eighteenth century, such was the interest which the subject had excited, and such the zeal with which it was prosecuted, that no less than one hundred and fifty-eight works appeared, or were issued from the press in different parts of England, and on the continent of Europe.

Were we permitted from the view that has been taken, to draw any inferences respecting what *has* been done during the present century, so far as it has past, and of the future, from facts that are well known and established, we hazard nothing in saying that the nineteenth century at its close, will probably exhibit a far greater amount of knowledge of the subject, far more extensive researches, and laborious efforts to explain and comprehend some of the hidden mysteries not yet understood, than has appeared at any preceding period of time.

More than half of the *number* of works that were published in the last century have already appeared, and numbers, as well naturalists as medical practiciens and dental surgeons, are engaged in microscopical investigations, with a view to explain and establish, if possible, every physiological and pathological fact that is embraced in this branch of medical science, or that can be interesting to the medical philosopher, the naturalist, or the dental surgeon. Of such is Professor Rhetzius, of Sweden, Weber, Heidlebrand, Schreger, Wagner, Parkinja—Professor Owens, of England, Blandin, Nasmyth, of London, and others in France, and different parts of the continent of Europe.

From these facts, is it not evident that this branch of science is not only advancing, but that it has fully kept pace with *every other* branch of the medical science?

We would, in the next place, direct the attention to the mechanical and operative department of knowledge, of which, although *not in strictness* an indispensable requisite qualification of a dental surgeon, nevertheless, it constitutes an essential part of the profession, as it has hitherto been, and is at present generally practised.

In order to a full and correct understanding of the origin and progress of this branch of the profession, it would be necessary to carry our views back to the days of the ancients, when Ovid, in swelling measures, attuned the lyre to gratify his muse. But this is by no means compatible with our limited time. If we commence a review of the time of the invention and introduction of porcelain teeth by D. Chement Dubois at Sevres, and especially the time of Fonzi's improved mineral teeth, and fixtures for obviating the partial or total loss of teeth, and which were considered, at the time, as unequalled in any part of the world, the improvements have been astonishing, both in Europe and America. We have had frequent opportunities of seeing examples of both, and we are free to say, that it appears difficult, nay, almost impossible that any further advances or improvements can be made, that may be considered a nearer approximation to perfection, than that which it has already attained, *especially in this our own country.*

In the early part of this discourse we claimed, as of right what custom and general usage had long since awarded us, viz. the title of efficient cultivators of an important branch of medical science.

In most foreign countries, those who have directed their attention to the study and practice of this branch of medicine, are known as such by the medical faculty, and have been recognized under the legitimate and appropriate title of dental surgeons.

Those whose diligent attention and talents were devoted to the acquirement of a correct and critical knowledge, as well of physiology and pathology, as of practical operations, were considered by the faculty of medical practiciens, as cultivators in the same field, and whose opinions upon professional points were entitled to the most respectful deference, and whose skill was, in many cases, considered as essentially necessary as that of the general surgeon or physician.

This was particularly the case from the time of Fauchard, whose opinions respecting deep seated and obstinate diseases of the bones of the head and face were often consulted, and whose professional services were frequently called in requisition by the practiciens of surgery as well as that of medicine.

This custom we find was prevalent in the time and practice of the celebrated French surgeon Petite, and several of his cotemporaries; and of dental surgeons, Bourdet, Bunon, Tenon, and others; but more especially Jourdain, whose familiar knowledge and pre-eminent skill, distinguished him in the estimation of the medical faculty, as possessing talents of a superior order as a dental surgeon, and worthy of all confidence.

From the time of Jourdain to the present, professional dentists of this kind have not been wanting to give character and standing to the profession, and for which many of the medical faculty have evinced the most decided marks of deference.

The same spirit is evinced by the enlightened and intelligent of the profession, and the same degree of zeal is manifested throughout France, and several other parts of the continent, and such as affords an earnest, nay, a guarantee that this, our branch of the science, has hitherto not only sustained its character, but, encouraged and cultivated by the talents and influence of such as are at present engaged in the cause, its course is onward and upward with the general science, to the utmost attainable limits of perfection.

Let us, for a moment change our ground, and contemplate the state of our profession, not as dentists, in the common acceptation of the word, but of the branch of the medical science, which we cultivate, as it has, and still exists in Great Britain, in order to a comparison with that of France, and that of our own country. By this means we shall be able to form a correct estimate of the improvements that have been made by members of our profession in other countries, in a given time, and in that of our own.

In the year 1770, Thomas Berdmore wrote and published a treatise on the teeth, a work as justly entitled to pre-eminence in point of merit, as any one that had ever appeared in the English language. His standing in his profession was such as to obtain for him the confidence of his rightful sovereign, George the III. to whom he was preferred as "dentist in ordinary"—in the early part of his reign, and for ought he knew to the contrary, as long as he lived.

The preference bestowed upon Mr. Berdmore, together with the honour conferred at the same time, would naturally operate as a stimulus to exertion, on the part of others who may have been engaged in the same pursuits, under the expectation, or at least the *hope* of succeeding to the honour, and sharing the emoluments most probably enjoyed by Mr. Berdmore, in case his situation should be vacated.

But whatever exertions may have been made to promote or advance the profession by those who were, at the time, engaged in it; or whether, in fact, Mr. Berdmore himself was ever engaged in any efforts to inculcate a more extensive and correct knowledge of the natural history of the teeth, and of the various diseases incident to them and the neighbouring parts, we have not the means of ascertaining. We believe, however, that we are safe in asserting that, although a number of medical writers had been engaged in an investigation of the natural history of the teeth, long anterior to this period, no practising dentist, not even Mr. Berdmore, had ever endeavored to enlarge the sphere of his knowledge beyond the mere mechanical operations incident to the profession; nor are we aware that any efforts were made by any English writer, expressly upon this branch of the medical science, until undertaken by Mr. John Hunter, in the year 1771.

We may here pause a moment, and examine, with advantage, the difference in the state of the profession, as to both theoretical as well as practical knowledge, in England—in France—and in several parts of the continent.

In 1770, as we have remarked, Mr. Berdmore wrote and published his treatise on the teeth. In 1728, *forty-two* years previous, Fauchard had published his memorable works. From that period, to the time at which Berdmore wrote, a number of valuable works on the dental science appeared on the continent, viz: Bunon in 1723—Mouton in 1746—Lecluse in 1755—Bourdet in 1758—Bunon again, in 1759—and Jourdain's celebrated work in 1766—besides a number of others of nearly equal merit.

About the time that Berdmore wrote his practical treatise, Mr. Hunter was engaged in his work on the natural history of the teeth, viz: 1771. From that period, to 1798, the year in which Mr. Blake wrote on the natural history of the teeth, no other attempt was made that we know of, to enlarge the sphere of knowledge in this branch of science; nor until 1806, eight years subsequently, did Mr. Fox contribute his share to the general fund of that kind of knowledge, so essentially important and necessary to assist those who were practically engaged in operations so intimately connected with the comfort and welfare of countless millions.

Were we now required to assign a reason why the dental science, now so called, should have remained so long, and so far behind that of the same profession, in a number of the large cities on the continent, we might assign several; but we know of none so likely to operate as a check or hindrance, to a successful prosecution of researches in physiology and pathology, as, amongst others, that of a want of encouragement on the part of *those* whose *duty* it was to give countenance and encouragement to such as would, most readily have engaged in efforts of this kind for the special benefit of medical science generally.

And no one, perhaps, is more chargeable with this fault than Mr. John Hunter, who, when he wrote his "practical treatise on the diseases of the teeth," &c. in 1778—instead of encouraging practical dentists in the prosecution of physiological and pathological researches and experiments, with a view to the further development of the latent phenomena of this branch of the science, as it was supposed his writings were expressly intended for, he threw a kind of hindrance—a negative prohibition in the way, which was calculated to check the ardour of scientific research, by saying—"the diseases which may arise in consequence of those of the teeth, are various; such as abscesses, carious bones, &c. many of which, although proceeding *originally* from the teeth, are *more* the object of the *surgeon* than the dentist, who will find himself as much at *a loss* in such cases, as if the abscess or carious bones were in the leg or any other distant part." And further—"all the diseases of the teeth which are common to them, with the other parts of the body, *should* be *put* under the management of the physician, or surgeon,—but those which are *peculiar to the teeth,* and their connections belong properly to the dentist." Again—"I shall, therefore, confine myself to the diseases of the teeth, gums, and alveolar processes; which parts having a peculiar connection, their diseases fall properly within the province of the dentist. I shall, also, *purposely avoid* entering into common surgery; not to *lead* the *dentist beyond* his depth, and to matters of which, it is *to be supposed,* he *has not* acquired a *competent* knowledge."—Hunter, on the natural history of the teeth, pp. 2 an 3, intro.

Very kind, and very considerate, indeed, in Mr. Hunter, to avoid leading the cultivators of an immediate branch of his own profession beyond *their depth,* or into error! Kind, indeed, in Mr. Hunter, to avoid leading his countrymen beyond *his depth,* and into *matters* of which, it is to be *supposed,* he has not acquired a competent knowledge. Why, if Mr. H. would have condescended to have cast his eye across the channel, amongst his frog-eating neighbors, he would there have found the works of those whose names I have enumerated, and especially those of Jourdain, who was not afraid to lead the dentists of his country beyond their depth; but who, twenty-two years before, had written and published a work, in comparison with which, Mr. Hunter's natural history of the teeth, dwindles into insignificance—a work replete with valuable instruction, and which every surgeon, physician, and dental surgeon, in Europe or America, may read with great pleasure, as well as advantage, and which *every* professional man ought to possess.

Little did Mr. Hunter imagine that the very language which he was using to deter the dentists from overstepping the bounds of their immediate province, by attempting the treatment of cases, which not understanding, they were not competent to, that his very language would have the effect of stimulating them to exertions that have resulted in the rearing of a Blake—of a Fox—and a host of others, naturalists and dental surgeons, down to Rhetzius, Owens, Nasmythe, and many others, with whom, by reason of their professional acquirements, and knowledge in dental surgery, the first and most eminent medical men on the continent of Europe, and in the British dominions, are now free to associate with, and to *consult* in many cases, especially such of a professional character as are of an obscure or anomalous nature.

Thus, while the light that was shed from the pen of Fauchard, was being diffused over France and many parts of Europe, Mr. Hunter's talents were brought into action to arouse the spirit of inquiry amongst the dentists in England, who, in a few years elevated the profession to that degree of importance and usefulness which is now acknowledged and appreciated in almost every civilized community in the world.

Let us now, for a moment, withdraw our attention from those who have been, and are still labouring in the cause abroad, and examine the state and condition of the profession in this, our own country.

Here is a field of a vast extent, and of sufficient importance to engross the pages of an ample folio. Strange as the subject may seem, and improbable as it may appear, it is, nevertheless, true, that, although the dental profession had been long known and practised in Europe, and many valuable volumes had been written on the subject, and published to the world, little or nothing was known of it in this country until about the year 1780, or until long after Mr. Hunter's work had been published. For, although his work on the natural history of the teeth was published in 1771, and his second part, on the diseases of the teeth, in 1778, scarcely a copy of the work was to be obtained, or to be found in this country, except in the possession of some American physician, who, on his return from Edinburg, where he had been to complete his medical studies, had supplied himself with a copy. Two or three copies of Berdmore, a plain, but valuable practical treatise, likewise found their way to this country, through the same, or a similar channel. I know of but two copies that were brought to this country during the revolutionary war, and they, like those of Hunter's, were retained in the private libraries of those gentlemen who purchased them in England for their own private use.

In this state of things, the few who were engaged in performing the common operations in practice, (and but few others, especially of a difficult nature were attempted) were groping their way in comparative darkness, and but for some fortuitous circumstances, it could scarcely have been considered otherwise than as a state of palpable ignorance of every circumstance that was necessary to connect the profession of a dentist with the science of medicine.

The first hints that were afforded, or opportunities offered to any person to obtain a knowledge of the profession, was, we believe, through a French dentist, by the name of Le Maire, who offered his services to the public during the revolutionary war. We do not pretend to a correct knowledge of his history, or of the manner in which he found his way to this country; but believe that it was with the French troops, who came to our assistance in our revolutionary struggle. However that may be, he had probably acquired a knowledge of the profession in his own country, where it had long been cultivated, and was not without some pretensions to skill in practical operations, especially in transplanting of teeth from the mouth of one person, to that of another, and by which, as he frequently performed the operation, he enjoyed a very lucrative business. He, likewise, undertook to instruct some two or three persons in the profession, which may be considered as the origin or commencement of dentistry (vulgarly so called) in this country.

Nearly about the same time, or not long subsequent, a rage for theatrical performances brought a company of foreign performers to this country, one of whom, a gentleman of polite address and accomplished manners, by the name of Whitelock, was a dentist, and who had probably acquired a knowledge of his profession in England.

From these sources, (and some others which we shall have occasion to mention,) it may be said our profession derived its existence, and for some years was gradually advancing in importance and usefulness, in proportion as talent and skill were evinced on the part of those who were engaged in the practice. But almost from the first efforts that were attempted in the practice, the whole course of proceedings was as perfectly mechanical as that of any other business. Not a ray of the light of science had as yet shed a gleam upon the subject, that could give it the colour, and much less a claim to any kind of relationship to the science of medicine. Not until an opportunity was afforded to some one of the profession to peruse the works of Mr. J. Hunter, did the light begin to dawn upon the subject, so as to awaken a conviction that the object of pursuit was based upon and intimately allied to science, and constituted an important branch of that of medicine.

This, it is believed, was long anterior to the year 1790, about which time, and in fact *long before*, much had been written and published on the continent, but as it was all in a different language from our own, and the intercourse at that time very limited, but little if any material advantage to the new adventurers in the profession was derived from that source; and but for a chain of fortunate circumstances, we might have been left to feed upon the faulty, nay, *very* erroneous opinions inculcated by Mr. Hunter, to this day. The circumstances referred to are of the following nature, viz.—Amongst the physicians that emigrated to the French West India colonies, and particularly Hispaniola, were some, at least, who when about leaving their own country, were careful to select amongst their medical works, such as were likely to prove useful in every department of surgery as well as the practice of medicine.

Amongst them were some of the best works on the treatment of diseases of the mouth and teeth. When the unfortunate disturbances, which threatened the peace and quietness of the people of that ill-fated island, began to assume an alarming aspect, the physicians, as well as all others that could avail themselves of the opportunities offered, fled with their effects to this or some other country for safety. Amongst the medical works thus saved were some of the most valued authors on dental science, and which were submitted to the perusal of those who were alive to the importance of the subjects which they contained, and who were ready to devour their contents with an eagerness seldom evinced by a half-famished gourmand.

These works were replete with not only new and valuable information on professional subjects, but they referred the reader to other equally valuable authors, which were, as soon as practicable, sought for and obtained.

From these circumstances, and from this period we may date the epoch when a mass of light, which before had been, as it were, long obscured by an *impenetrable* cloud, burst upon the comparatively few votaries of our profession in this country, with an almost overwhelming effect, and soon began to awaken them to a sense of the low and inferior grade which they were occupying in the scale of dental surgeons; and to convince them of the necessity of still higher attainments and more exalted qualifications to entitle *them* to a rank amongst the zealous cultivators of an important branch of the healing art—I mean a more intimate and familiar acquaintance with our physical organization, or with physiology and pathology.

From the view that has been given, it is not to be supposed, and much less believed, that the profession had remained in a stationary condition from the time of its first introduction into this country to the time above alluded to. The lessons that were inculcated in the first instance, by foreigners, were deeply impressed, and there were soon found amongst us examples of native genius and talent, that were congenial with the most encouraging as well as successful culture.

In adverting to a few instances of this kind, we shall purposely leave out of view the living examples, and offer, with feelings of great personal regard, and with sentiments of deep professional deference, the justly merited tribute of respect to those whose names we take pleasure in enrolling amongst those who, in reality, were the efficient practiciens, and promoters of dental science in this our country—but whose names, whose talents, and whose exemplary characters are all that is left us to respect and admire; and more especially to imitate.

Amongst those of native origin, we shall mention the name only of Mr. J. Greenwood; a sketch of whose biography is to be found in the Journal of Dental Science. In early life, it seems he, like many of the sons of New England, evinced an energy of character, which encouraged and sustained him through the vicissitudes of the remaining part of his life.

Besides this essential quality, he was endowed with no ordinary share of ingenuity and mechanical tact. In this opinion we are sustained by the fact, that he must have commenced his professional career in the city of New York, about the year 1788 or '90; at the latter of which periods, we believe he stood alone in his profession, enjoying almost the exclusive patronage and confidence, not only of the inhabitants of that city, but of the *Father of his country,* George Washington, himself, and for whom he performed an operation, and executed an entire dental apparatus, which for ingenuity and mechanical skill, would have done credit to the most experienced of the profession in any country; and the more so, as we have reason to believe that it was the first attempt of the kind that had ever been made in this country; and, moreover, that he had *never seen* an example of the kind, either by a drawing or otherwise, to serve him as a model or guide. This specimen we have seen in his own office, and examined in his own hands.

This, from the novelty of the case, and from the very prominent standing and reputation of the person for whom it was performed, was sufficient to ensure to Mr. G. the confidence of the public—to establish his character and reputation as a dentist; but, moreover, to give to the profession an eclat that spread far and wide, and prompted many half desponding advocates for a *renewal* of long lost *juvenile attractions,* to resort to the toilet, and there to deliberate upon the sad alternative of submitting themselves to the *reputed tortures* of the dentist's mill, to be ground over, and made young again, at least in appearance.

Suffice it to say, (and that without levity,) that from his time, the advances by valuable improvements, in practical dental operations, were onward and rapid; and that too, by unlooked for and valuable auxiliary aid; for about this period, the importance and respectability of the profession was considerably enhanced by the auspices of the well known talents and abilities of the elder Mr. Gardette, who, after having obtained a valuable fund of professional knowledge, before leaving his own native country, (France,) came and settled in Philadelphia, where he soon experienced the most flattering encouragement for his services, and ample field for a display of his mechanical and professional skill.

Previous to, or about the time of Dr. Gardette's successful practice, a Dr. Spence, of Philadelphia, was likewise enjoying considerable confidence and reputation in his profession, as a dentist. With his commencement, or advances in skill or capacity, we have not been able to obtain as clear and satisfactory account as we could have wished. We believe, however, that he obtained his first instruction in the business from a French dentist, already mentioned, by the name of Le Maire.

At no distant lapse of time, the profession experienced another no less valuable, nay, very important accession of professional skill and talent, in the arrival of the late Dr. Edward Hudson, from Dublin, who likewise preferred Philadelphia for his future residence, and professional practice.

From such an unexpected, yet seasonable addition of acknowledged talent and respectability, it would scarcely fail of enhancing the value, and of ensuring the celebrity of the profession, and of its utility and importance to every class of society. Nor did the profession fail to experience this effect; nor was the public disappointed in its expectations.

The light that was now dawning upon us seemed to operate as a stimulus to exertions for preferment in excellence in other parts of our country; and which soon experienced an additional impulse, by the offer of the professional services of a Mr. Woofingdale, who arrived in New York about the year 1794 or '5, direct from the metropolis of England, and with strong recommendations for skill and respectability of character.

Thus, while the profession was dispensing its benefits and usefulness to the northward and eastward, through the diligent and successful practice of Messrs. Greenwood and Woofingdale, and some few others, the profession was advancing with rapid strides to usefulness and respectability, by the skill and ingenuity of Messrs. Gardette and Hudson, of Philadelphia, who were dispensing the benefits and example of their *superior* skill and professional talents to the west and the south, where they were at all times considered as worthy of imitation, and were even sought after with that view, and for that purpose; and when an opportunity of the kind was met with, it seemed to exercise a contagious influence upon such as were disposed to acquire a knowledge of the various operations upon the teeth necessary to constitute a dentist, in the estimation of a few only, at that period of time. In this way, or by these means, the number of nominal dentists has greatly increased, until from the facilities with which a smattering of the subject was obtained, the country, as at present, became overrun with empyrics, and unblushing pretenders.

We have thus far, and in this brief and imperfect manner, endeavoured to give some general ideas of the origin of our profession; of its gradual development; of its increasing importance, as an art, and of its wide spreading usefulness in foreign countries; but more especially in our own. That for a great length of time, it was considered in no other light than as a pursuit purely mechanical, and that the success in business depended very much upon the skill and address of the operator. And although in its nature and application, connected with, and involving *organized structures*, and living complex tissues, little or no attention was paid to the fact, nor was it considered necessary that a person engaged in practice, should have any knowledge of the anatomy of the parts to be operated on.

The time arrived, however, when this state of things was no longer to be tolerated. The profession was found to involve principles and parts connected with those of anatomy; and that it could not, nor ought not to be considered in any other light than as a *branch* of the medical science; hence it was treated as such by Fauchard and most of the subsequent writers on the natural history and diseases of the teeth.

We have remarked, that a knowledge of this fact was early seen, and attended to by several of the profession, and with so much and so decided advantages as to attract the attention, and merit the approbation of the medical faculty; who readily saw, and as readily encouraged the cultivation of this branch of medicine, as intimately connected with the general science, and as affording results highly beneficial to the general science, particularly in obstinate neuralgic affections growing out of, or depending upon, long continued local chronic inflammation in the neighbouring parts.

This view of the subject held out strong encouragement to such as stood prominent as dentists, to cultivate a more familiar acquaintance with physiology and pathology, for in so doing he enjoyed more frequent interviews, and a more familiar acquaintance with practiciens of medicine, thereby gradually introducing and establishing a line of distinction between such as could be considered in no other light than as mechanical dentists, and such as were deservedly entitled to the appellation of dental surgeons.

While the mechanical department was advancing; while new and valuable improvements were daily being made in instruments, and in the treatment of the various cases that occur in the operative department, this spirit of physiological research was widely spreading, and generally prevailing on the continent of Europe, long before Mr. Hunter's work on the natural history of the teeth was published.

We have observed, that on the publication of this work, the light of dental science first began to dawn upon, and awaken the attention of the members of the profession in England. It was soon increased in intensity, and interest, by the publication of the works of Messrs. Fox and Blake, which were soon obtained and circulated amongst the members of the profession in this our own country, thereby promoting and encouraging the spirit of inquiry and research, while at the same time, a knowledge of the interesting works that had been published on the continent, were being made known, and their contents assisting greatly, to extend and diffuse a correct scientific knowledge of this branch of medical science, not only amongst the members of the profession in this country, but amongst those of almost every other.

This may be considered the period when the light shone in copious measures upon this branch of medical science, and which, though long known and cultivated on the continent, was generally diffused amongst the dentists throughout England and America; and that to such a degree as to inspire the confidence, and meet the approbation of most reputable members of the medical faculty, and to induce them to admit the justness of the claim of the dental surgeon to a participation in the honours justly due to each and every one whose attention was engaged in cultivating a *scientific knowledge* of this, as well as every other branch of medical science.

They were free to acknowledge the correctness of the views of those, who by study and practice had become familiar with this branch of medicine, and to treat with respectful deference the opinions of dental surgeons upon cases of an obscure or doubtful character that occurred in their practice. Nay, more! We announce the fact, not with any degree of vanity or exultation, but with a peculiar innate gratification, that an eminent professor of one of our universities, in recommending to a numerous class of students to attend a course of lectures about to be delivered on dental physiology and pathology, observed that "a knowledge of the subject was *essentially* necessary and useful to a practicien of medicine, but which had not been, hitherto, recognized or taught in the medical schools."

We have thus far attempted an imperfect sketch of the origin, progress, and present condition of our profession, or of that of the dental science, and however unimportant or uninteresting it may appear in the public estimation, may we not for a moment pause and ask, is this that branch of science, or profession, that has been sneered at, derided and denounced, as too contemptible for the contemplation and pursuit of any rational or reputable person? We answer, yes! Moreover, is it asked, is this the branch of that noble, that divinely sanctioned science, that nearly five hundred years before the christian era, had engaged the attention and study of a Hippocrates, and *ever since*, most of the medical philosophers and writers, down to our own American Hippocrates and philosopher, Dr. Benjamin Rush, who recommended the subject to the medical faculty of our own country, "as one that will admit of much *curious* and useful information?" We answer, O yes. And as such we commend it to every member of our profession, as highly worthy of their contemplation and their zealous cultivation; not agreeable to a prevailing custom with many of the profession of the present day, but agreeable to a course, which, if diligently pursued and attentively studied, will insure an elevation that shall command the respect, not only of every member of our own profession, but of that of the medical faculty in every part of the world.

## Figures and Tables

**Figure f1:**